# Terrain Traversability via Sensed Data for Robots Operating Inside Heterogeneous, Highly Unstructured Spaces

**DOI:** 10.3390/s25020439

**Published:** 2025-01-13

**Authors:** Amir Gholami, Alejandro Ramirez-Serrano

**Affiliations:** Department of Mechanical and Manufacturing Engineering, University of Calgary, Calgary, AB T2N 1N4, Canada; aramirez@ucalgary.ca

**Keywords:** traversability, unstructured spaces, point cloud processing, multi-legged robots

## Abstract

This paper presents a comprehensive approach to evaluating the ability of multi-legged robots to traverse confined and geometrically complex unstructured environments. The proposed approach utilizes advanced point cloud processing techniques integrating voxel-filtered cloud, boundary and mesh generation, and dynamic traversability analysis to enhance the robot’s terrain perception and navigation. The proposed framework was validated through rigorous simulation and experimental testing with humanoid robots, showcasing the potential of the proposed approach for use in applications/environments characterized by complex environmental features (navigation inside collapsed buildings). The results demonstrate that the proposed framework provides the robot with an enhanced capability to perceive and interpret its environment and adapt to dynamic environment changes. This paper contributes to the advancement of robotic navigation and path-planning systems by providing a scalable and efficient framework for environment analysis. The integration of various point cloud processing techniques into a single architecture not only improves computational efficiency but also enhances the robot’s interaction with its environment, making it more capable of operating in complex, hazardous, unstructured settings.

## 1. Introduction

Traversing confined and unstructured environments poses significant challenges in the field of robotics, particularly when dealing with varying geometric environmental conditions that demand high levels of adaptability and real-time decision-making. Autonomous robots require sophisticated perception and navigation systems to traverse these environments safely and efficiently. This paper introduces an advanced sensing data processing methodology centered on enhancing robotic perception to enable robots to identify critical terrain properties through new point cloud processing techniques. By integrating rotational adjustments, voxel-filtered cloud, and boundary and mesh generation, the approach not only refines environment modeling but also enriches the robot’s perception capabilities, enabling it to better interact with its environment. This methodology is for developing multi-legged autonomous systems capable of operating in diverse, complex settings from industrial landscapes (inside underground facilities) to natural disaster sites (hurricane-devastated areas).

Recent advancements in robotics have highlighted the importance of accurate and efficient terrain analysis for safe navigation [1,2]. Although the developed techniques work well in simple (structured) terrains such as stair-climbing [3] and stepping on non-coplanar, randomly positioned surfaces, they often fall short when dealing with the stochasticity and uncertainty inherently found in heterogeneous, unstructured environments. The proposed approach addresses these limitations by employing a new set of formulations (indexes) to enhance environmental assessment. A key to the methodology is the use of a voxel-filtered cloud, which reduces data redundancy and computational overhead while preserving critical spatial features. Additionally, advanced meshing techniques are utilized, such as alpha shapes, to generate effective mesh representations of the environment. These meshes are then analyzed to extract meaningful geometric features, such as slope and terrain roughness, which are vital for assessing the traversability of different (a priori unknown) environments.

To improve the computational processing time and facilitate the identification of occupied and empty spaces within a designated area, the concept of a virtual plane is introduced. This approach simplifies the analysis of 3D point cloud data by projecting relevant terrain features onto a 2D virtual plane placed in front of the robot’s direction of motion. The virtual plane, combined with an adaptive ellipsoidal boundary model, enables robots to determine how to traverse safely through the perceived/sensed cluttered and dynamic environments.

## 2. Literature Review

When robots operate in heterogeneous, real-world scenarios, they encounter environments with various geometrical (e.g., slope, valleys, and elevation changes) and physical (e.g., soil type, roughness, friction, and humidity) characteristics. Evaluating these environment characteristics and determining how robots can pass over them are crucial for determining if and how a robot can traverse the perceived environment. The concept of traversability, defined as a robot’s ability to cross a terrain region in an admissible state, has garnered significant attention in the robotics community [4,5,6]. Traversability methods are primarily divided into two categories: trainable and non-trainable methods [7].

### 2.1. Trainable Methods

Machine learning and deep learning techniques [8,9,10] have significantly contributed to environment perception and terrain assessment. These techniques have used diverse estimation tools to assess terrain traversability through machine learning approaches [11,12,13]. Given the high levels of uncertainty present in unstructured environments, researchers have employed traversability estimation techniques to generate elevation grid maps, which classify regions into traversable and non-traversable categories [14,15,16,17,18], providing critical information for robot path-planning and navigation [19,20,21]. However, such formulations often limit the number of aspects used to identify terrain traversability and fail to consider the robot’s motion and reconfiguration characteristics (which influence how a robot can traverse the perceived terrain). Therefore, we believe various levels of stochasticity/uncertainty need to be considered when evaluating a terrain for a specific robot.

In addition, previous mechanisms are naïve, assuming rigid terrains and neglecting long-distance perception and compliant surface traversability, which must be taken into account. Thus, there is a need to extend the current traversability formulations to include compliant and deformable terrains. Given that these terrain characteristics change continuously, assessing terrain in a consistent manner has proven to be challenging.

### 2.2. Non-Trainable Methods

To discretize the environment into more granular levels of traversable and non-traversable regions, researchers have employed techniques such as occupancy grid maps [22,23], elevation maps [24,25], and traversability maps [26,27]. Numerous studies have utilized these mapping frameworks to find reliable paths for robot traversal while avoiding collisions [14,15,19,20,28,29] and maintaining stability [30].

Traversability algorithms have been used to identify paths and corresponding footstep locations on given terrains [31,32]. To evaluate terrain characteristics, researchers have developed footprint traversability mapping algorithms based on elevation maps, which generate a traversability score that takes into account changes in the terrain’s height and slope [33,34]. These traversability maps have enabled robots to find feasible footholds [35,36]. Although these methods have equipped robots with the ability to traverse diverse 2D (planar) and 212D (rough) spaces, there is a need to enhance these algorithms for use in 3D confined, hazardous spaces, where terrain perception is typically compromised by the presence of obscurants such as dust, smoke, poor light conditions, and other obstructions.

Early attempts at traversability estimation have relied on basic obstacle detection algorithms using monocular color vision, classifying obstacles and terrains into discrete classes [37,38]. The traversable regions identified by these algorithms are, however, highly dependent on the robot’s movement, leading to numerous limitations.

Current methods often struggle with the inherent uncertainty and variability of unstructured environments, particularly in 3D confined spaces. These challenges necessitate the development of sophisticated techniques to accurately assess the characteristics of complex terrains positioned at diverse orientations around a robot. While trainable methods using machine learning and deep learning have advanced terrain assessment by generating 212D elevation grid maps and classifying traversable regions, they often fall short in analyzing terrains in full 3D space located on top of, beside, in front, behind, and above a robot. Current trainable methods do not include important aspects such as a robot’s motion characteristics or sensing limitations (e.g., sensor characteristics). On the other hand, non-trainable terrain assessment methods such as occupancy grid maps and elevation maps provide reliable information for path-finding but assume that only basic characteristics such as the location of obstacles are known, while disregarding the terrain properties that might affect the robot’s locomotion (e.g., slipping surfaces). Such mechanisms also ignore the continuous variability of the environment. The limitations present in both approaches highlight the need to develop robust algorithms that can handle the dynamic and stochastic nature of real-world 3D spaces.

To address these limitations, this paper proposes an innovative approach that integrates point cloud processing techniques to provide a more detailed and efficient environment analysis, thereby enhancing robotic perception and the ability to better determine robot traversability. The goal is to enable a reliable terrain process that evaluates key aspects of terrain traversability—slopeness, roughness, and the presence of empty spaces—using point cloud data. By leveraging these aspects, the proposed method offers a comprehensive framework for assessing and navigating complex environments. Researchers can utilize this algorithm to enable robots to dynamically adjust their routes, avoid obstacles, and navigate complex environments effectively.

## 3. Terrain Characterization Process

In this paper, three traversability indices—slopeness–roughness–empty space (SRE) indices—are proposed to provide a comprehensive evaluation of terrain, enabling robots to make traversal decisions about their present environment. These indices determine three basic terrain characteristics: slopeness (the steepness of the surface), roughness (the irregularity or ruggedness of the terrain), and the presence of empty spaces (areas where no solid surface is detected).

Even though the above-mentioned three terrain characteristics have been used in the past to determine how a robot should/could navigate perceived terrain, previously proposed mechanisms have only considered engineered or semi-engineered terrains such as ramps, carpets, and simple, granular terrains.

Here, however, the proposed SRE indices consider highly complex terrain geometries found inside collapsed buildings, forest fire zones, and disaster response operations, where sensor data tend to have diverse errors, data are difficult to collect (e.g., due to obscurants in the environment such as dust, smoke, and fumes), and terrains have diverse and heterogeneous geometrical (non-coplanar surfaces) and physical (e.g., friction) properties.

The SRE indices are utilized independently to provide the robot with essential information for determining how to traverse and which locomotion mode (e.g., walking or crawling) is best suited for the perceived terrain.

**Figure 1:**
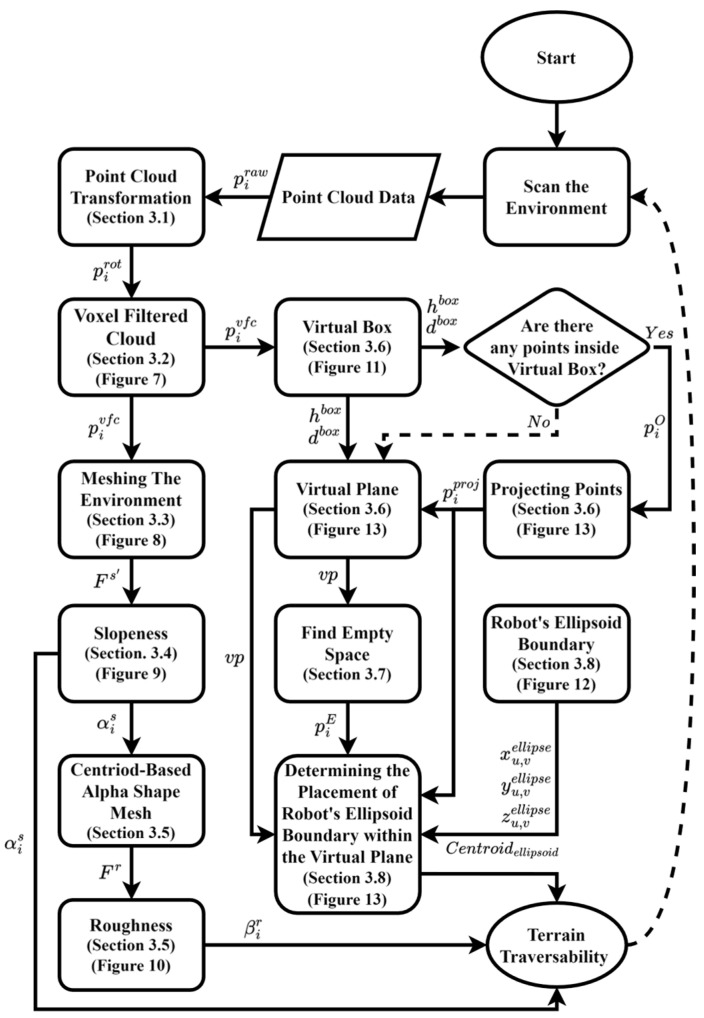
Flowchart of the proposed SRE process.

The process (Figure 1) begins by scanning the environment to capture the 3D point cloud data, $p_{i}^{raw}$. If needed, such point cloud data are transformed to align the sensed data (see Section 3.1) with the robot’s body frame of reference, $p_{i}^{t}$ (to account for potential misalignment between the sensor and robot’s body frame, e.g., when the head of the robot containing the sensor tilts and pans as the robot moves).

In sensors like LiDAR and depth cameras, point cloud data might contain millions of points per second. To reduce the processing time that might be needed to process all such data and address the noise of the 3D transformed point cloud, $p_{i}^{t}$, data undergo voxel-filtering (see Section 3.2). Voxel-filtering reduces redundancy and computational overhead by aggregating points into uniformly sized voxels, balancing detail preservation with processing efficiency. Following this, duplicate points are removed to create a concise and representative point cloud, ensuring spatial fidelity and identifying unique points in the set $P^{vfc}$. The voxel-filtered data undergo two separate processes: meshing the environment (Section 3.3) and defining a virtual box, $h^{box}$ (Section 3.6).

A 212D (surface) mesh, which is a set of vertices $V^{s}$ and faces $F^{s}$ representing the environment, is created from the voxel-filtered data (e.g., tetrahedrals). The mesh is further refined using a filtering process, where only faces with areas within a specified threshold $T_{A}$ and side lengths within threshold $T_{L}$ are retained, forming the refined set of faces $F^{s^{\prime }}$, leading to a more efficient representation of the environment.

After meshing the environment, the refined meshes allow for slopeness calculation, $\alpha_{i}^{s}$, by extracting normal vectors at each mesh element and comparing them to a horizontal reference plane. This process quantifies the terrain’s steepness using trigonometric calculations (Section 3.4).

Subsequently, the centroids of refined mesh faces $F^{s^{\prime }}$ are used to construct a centroid-based alpha-shape mesh, which is a set of faces, $F^{r},$ to calculate surface roughness, $\beta_{i}^{r}$ (defined as local variations and irregularities in the surface geometry), thereby enhancing the understanding of the terrain characteristics, as described in Section 3.5.

**Figure 2:**
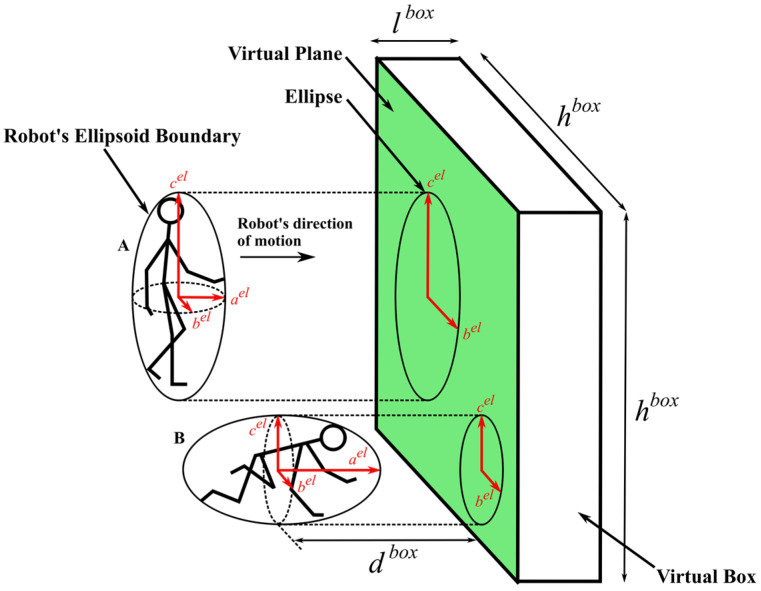
Projection of the robot’s ellipsoid boundary onto a virtual plane. A: Robot in a walking state. B: Robot in a crawling state.

Simultaneously, a virtual box, a conceptual and dynamically defined boundary in 3D space positioned in front of the robot at distance $d^{box}$ with dimensions of height $h^{box}$ and depth $l^{box}$, is defined within the environment to identify the voxel-filtered terrain’s data points that lie inside it, limiting the area of interest for data processing and analysis. The distance between the robot and the virtual box can be determined (modified) as needed. If points are found, they are projected onto a virtual plane, vp, which is created by projecting the virtual box onto its front face—the side of the virtual box that faces the robot, as shown in Figure 2. The purpose of the virtual plane, defined in Section 3.6, is to identify non-traversable (occupied spaces) and traversable (empty spaces) space in front of the robot, helping it determine how to safely traverse the area.

To determine if the robot can traverse through empty spaces while avoiding occupied spaces within the virtual plane, the robot’s geometric structure is defined by an ellipsoidal boundary. The robot’s ellipsoidal boundary encapsulates its geometric structure, providing a mathematical representation that outlines the robot’s shape as an ellipsoid—a three-dimensional geometric figure. This representation simplifies the complex shape of the robot into a more manageable form, enabling a clear depiction of its physical dimensions. The characteristics of the robot’s ellipsoid boundary, including its axes, are determined based on its kinematics, motion state, and direction of motion.

Considering the virtual plane and projected points $p_{i}^{proj}$ within it, the robot’s ellipsoid boundary is also projected onto the virtual plane. The robot’s ellipsoid projection on the virtual plane, denoted as an ellipse, represents the free space area needed by the robot to move through the environment following a given locomotion modality (e.g., walk, crawl, etc.), as illustrated in Figure 2.

The flowchart in Figure 1 illustrates the process of showing how point cloud data are utilized to assess terrain traversability. The following sections provide a detailed description of each part of the flowchart.

### 3.1. Point Cloud Transformation

For a robot to function effectively, it requires an accurate perception of its environment. Sensors like LiDAR and depth cameras capture detailed spatial information, but the raw data they generate are often misaligned with the robot’s base coordinate system due to the independent motion of various body parts. When a sensor mounted on a robot begins scanning, it captures point cloud data in the sensor’s reference frame. However, for the robot to utilize these data effectively, such as for meshing the environment, there is a need to transform the sensor data from the sensor frame to the robot’s base frame. Given that the robot’s body parts may move, the sensor’s position and orientation will change relative to the robot’s body base over time. To account for these dynamic changes, a homogeneous transformation matrix is employed to transform the sensor data into the robot’s base frame of reference. This homogeneous transformation matrix, T, is derived from the robot’s kinematic chain, utilizing a framework similar to that described in [39], and accounts for both rotational and translational movements along the X, Y, and Z axes. To ensure continuous updates of the robot’s orientation in 3D space, the roll, pitch, and yaw data from an Inertial Measurement Unit (IMU), mounted beneath the robot’s base within the kinematic chain, are integrated. The IMU mounted beneath the robot’s base is the VectorNav VN-200, which provides roll, pitch, and yaw (attitude) estimates through the onboard Extended Kalman Filter (EKF). In GPS-denied environments, the VN-200 primarily relies on its magnetometer for heading (yaw) estimation, achieving an accuracy of 2.0° RMS, while pitch and roll, aided by the accelerometer, have an accuracy of 0.5° RMS.

Let $P^{raw}=\left\{p_{i}^{raw}\right\}$ represent the point cloud data obtained from sensor readings, where each point $p_{i}^{raw}=\left(x_{i}^{raw},y_{i}^{raw},z_{i}^{raw}\right)$ denotes the Cartesian coordinates of the i-th point in the sensor’s reference frame. Using Equation (1), each point $p_{i}^{raw}$ is transformed from the sensor’s reference frame to the robot’s base frame. To apply the homogeneous transformation, each point $p_{i}^{raw}$ needs to be converted into homogeneous coordinates by appending a fourth dimension, resulting in $p_{i}^{raw}=\left(x_{i}^{raw},y_{i}^{raw},z_{i}^{raw},1\right)$.

(1) $$p_{i}^{t}=T.p_{i}^{raw}$$

Here, T is a 4×4 matrix and applies the homogeneous transformation matrix to the $p_{i}^{raw}$ to obtain the transformed point $p_{i}^{rot}$ in the robot’s base frame.

### 3.2. Voxel-Filtered Cloud

The transformed point cloud data $p_{i}^{t}$ acquired in Section 3.1 is subject to noise and errors originating from various sources, such as sensor inaccuracies, environmental factors (e.g., weather conditions), reflections from shiny surfaces, and motion artifacts. Therefore, an efficient point cloud filtering technique is essential to extract relevant data points and remove unnecessary ones, which, in turn, reduces computational costs in subsequent processing steps.

The proposed filtering technique improves the efficiency of point cloud data processing by using a two-step filtering technique designed to refine data while preserving critical spatial features. These steps include voxel-filtering of the transformed point cloud and the removal of duplicate points.

Voxel-Filtering: This step reduces redundancy and computational overhead by dividing the 3D space into uniform-sized voxels. Points within each voxel are aggregated, reducing redundancy while preserving the essential structural information of the environment. This step decreases the computational demand of subsequent processing steps.

Mathematically, the coordinates of each transformed point $p_{i}^{t}$ are quantized to the nearest voxel size $v_{size}^{map}$, effectively partitioning the space into discrete voxel units (Equation (2)).

(2) $$p_{i}^{map}=round\left(\frac{p_{i}^{t}}{v_{size}^{map}}\right)\times v_{size}^{map}$$

Equation (2) governs the reduction in data density while preserving spatial integrity. By applying voxel filtering to the transformed point cloud, each point $p_{i}^{t}$ is assigned to a specific voxel coordinate $p_{i}^{map}$, corresponding to its position in the 3D space after rounding. The voxel size $v_{size}^{map}$ determines the granularity of the downsampling process, balancing data resolution and computational efficiency; a smaller voxel size preserves more detail but requires more computational resources, while a larger voxel size reduces detail but saves computational resources.

Removal of Duplicate Points: Following voxelization, removing duplicate points within each voxel ensures a concise and representative point cloud. The objective is to identify unique points within the set $P^{map}=\{p_{i}^{map}\}$. These unique points are defined as a set that contains only distinct elements, which we denote as $P^{vfc}=\{p_{i}^{vfc}\}$.

Efficiently filtering and refining the point cloud data results in a more accurate and concise representation of the environment, which is essential for the subsequent step: meshing the environment.

### 3.3. Meshing the Environment

Recent developments in 3D scanning technologies have produced extensive point cloud datasets, capturing detailed spatial information. These datasets are valuable for applications such as 3D modeling, environmental mapping, and boundary detection. However, extracting meaningful boundaries and generating structured mesh representations from point clouds is challenging due to noise, irregularities, and sparse data distribution. Existing techniques, such as occupancy grids [40,41,42], often struggle in complex environments with irregular obstacles and fine geometrical details. This is primarily due to their fixed grid resolution, which can fail to capture small, intricate features and gaps, especially in unstructured terrains. Moreover, due to the potential deployment of the robot in diverse environments, including complex settings and disaster scenarios, establishing ground truth, precise reference data used to validate algorithm performance is not feasible. This is due to the dynamic and irregular nature of these settings, where terrain can change unpredictably, and the absence of pre-existing maps or models makes it impractical to gather accurate reference data.

Alpha shapes provide an alternative approach that efficiently captures the topology of point clouds. This methodology consists of several stages, from data preprocessing to boundary detection and mesh generation. The core process involves computing the alpha shape from the preprocessed point cloud data. Alpha shapes are based on computational geometry, which defines meaningful boundaries based on the local density and point distribution. In this approach, one can identify the major structural features within the dataset in order to have it analyzed and interpreted. 

Prior to applying the alpha shape algorithm, the input point cloud data undergo preprocessing to remove noise and irrelevant information. The aim of preprocessing data is to enhance the quality of the data and improve the accuracy of the resulting mesh, as discussed in Section 3.2. Alpha shapes offer a geometric framework for capturing the shape of a point set, emphasizing both convex and concave regions based on a user-defined α parameter [43], with smaller values producing tighter boundaries and larger values resulting in more relaxed shapes.

Consider a set of points $P^{vfc}$, which is a finite set in $R^{3}$. To understand the topology of these points, the alpha shapes method is employed, which relies on the geometric framework of Delaunay triangulation DT. DT is the triangulation of $P^{vfc}$, such that no points in $P^{vfc}$ lie inside the circumcircle (in 2D) or circumsphere (in 3D) of any simplex ∆ (triangle in 2D, tetrahedron in 3D) in the triangulation. The simplices that construct DT can be points, line segments, triangles, and tetrahedra. So, *DT* is obtained as the collection of simplices.

The next step in the construction of the alpha shapes involves calculating the circumcenter and circumradius for each simplex ∆. The circumcenter c(∆) is the center of the circle or sphere that passes through all vertices of ∆ and the circumradius is the radius of that circumcircle or circumsphere. Considering these calculations, an α-ball $B\left(c\left(∆\right),\alpha \right)$ is defined as centered at the circumcenter c(∆), with radius α.

A simplex ∆ is considered part of the alpha shape, i.e., α-exposed if the following conditions are satisfied:

- *r*(∆) ≤ α;
- The α-ball B(α) should not contain any points from $P^{vfc}$ other than the vertices of ∆, i.e., *B*(*C*(∆), α) ∩ *P^vfc^* = {*vertices of* ∆}.

When these conditions hold true, the simplex ∆ is termed α-exposed, and it contributes to the formation of the alpha shape. The alpha shape is then constructed as the union of all α-exposed simplices $U\left(\alpha \right)$ (see Equation (3)). This union represents the shape that emerges from the point cloud at the given scale α, capturing the essential features of the environment defined by the points.

(3) $$U\left(\alpha \right)=\overset{n}{\underset{i=1}{⋃}}B_{i}\left(c\left({∆}_{i}\right),\alpha \right)$$

where $B_{i}\left(c\left({∆}_{i}\right),\alpha \right)$ represents the α-ball for the i-th simplex ${∆}_{i}$, centered at $c\left({∆}_{i}\right)$ with radius α. n is the total number of simplices being considered.

The boundary of the alpha shape is formed by the faces, edges, and vertices of these simplices that are not shared by any other α-exposed simplices.

The parameter α is crucial in controlling the level of detail in the resulting shape. By constructing Delaunay triangulation meshes, the algorithm removes edges exceeding the alpha radius, where α is a real number within the range 0≤α≤∞. The influence of various α values is outlined below.

- α → α: The alpha shape becomes the full Delaunay triangulation, ultimately forming the convex hull of *P^vfc^*.
- As α decreases, the alpha shape becomes more intricate, preserving finer features and capturing concavities of the point cloud.
- α = 0: The alpha shape is just the set *P^vfc^* itself.

**Figure 3:**
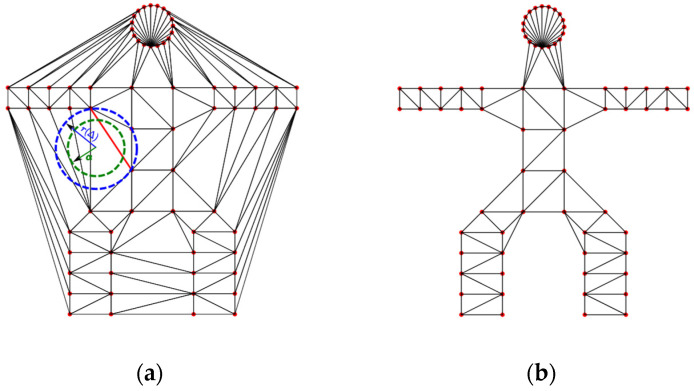
Visualization of the alpha shape construction process for a point set representing a simplified human figure (T-pose) with α=0.8. (**a**) Delaunay triangulation. (**b**) Alpha shapes.

Figure 3a shows the Delaunay triangulation of a point set (represented by red dots), with a circumcircle $r\left(∆\right)$ (dashed blue line) and an α-ball with radius α (dashed green line) shown as an example. In this figure, since $r\left(∆\right)>\alpha$, the simplex ∆ (red line/edge) is not considered part of the alpha shape and, thus, the edge is removed. This procedure continues iteratively until the final alpha shape is constructed. Figure 3b displays the final alpha shape boundary formed by the edges of the α-exposed simplices. The alpha shape captures the overall structure of the point set while smoothing out finer details, demonstrating the influence of the alpha parameter on the resulting shape.

Despite their advantages, Alpha shapes have limitations in detecting surface features because no single alpha parameter can effectively include all desired triangles while excluding undesired ones. They often struggle to distinguish between actual surface points and points marking the edge of breaks, leading to incorrect inclusions. Additionally, when two separate objects are close together, alpha shapes may mistakenly connect triangles from both surfaces. In regions with sharp turns or joints, they often produce a “webbed-foot” appearance, failing to accurately represent the intended geometry. These issues can negatively affect the quality and usefulness of the generated mesh in various applications [44].

To address these limitations, post-processing is employed following the application of the alpha shape algorithm to refine the mesh boundaries. This process involves discarding irrelevant segments and focusing on accurately defining obstacle boundaries. During post-processing, each face (triangle) in the mesh is evaluated based on its triangle area and side lengths. The area of a triangle is computed using the cross product of two vectors formed by its vertices, while the side lengths are calculated using the Euclidean distance between the corresponding vertex pairs. Only faces that meet specific geometric criteria—where the area or any side length exceeds a user-defined threshold—are retained. This step effectively removes non-representative faces, resulting in a refined mesh that more accurately captures the underlying geometry of the environment.

Given a mesh represented by vertices $V^{s}$ and faces $F^{s}=\left\{f_{i}^{s}\;|\;i=1:n\right\}$, where each face $f_{i}^{s}$ is defined by three vertices $v_{ij}^{s}$ (with j=1:3), we propose a filtering process that yields a refined set of faces $F^{s^{\prime }}\subseteq F^{s}$. In simpler terms, a face $f_{i}^{s}$ is retained if either its triangle area $A_{i}^{s}$ is within a designated threshold, $T_{A}$ (as shown in Equation (4)), or if any of its three side lengths $L_{i}^{s}$ are within a designated threshold, $T_{L}$ (as shown in Equation (5)). This filtering process ensures that only relevant faces are retained, contributing to the refinement of the mesh data.

(4) $$A_{i}^{s}=\frac{1}{2}\left|\left|\left(v_{i2}^{s}-v_{i1}^{s}\right)\times \left(v_{i3}^{s}-v_{i1}^{s}\right)\right|\right|;\;{\;A}_{i}^{s}\leq T_{A}$$

(5) $$L_{i}^{s}=\frac{1}{2}\left\|\left(\begin{array}{c} v_{i1}^{s}-v_{i2}^{s}\\ v_{i1}^{s}-v_{i3}^{s}\\ v_{i2}^{s}-v_{i3}^{s} \end{array}\right)\right\|;\;\;{\;L}_{i}^{s}\leq T_{L}$$

The selected thresholds $T_{A}$ and $T_{L}$ are determined through a trial-and-error method, testing various values to effectively retain meaningful geometric features. This iterative approach allows for adjustments based on empirical analysis of the resulting mesh quality.

### 3.4. Slopeness

Once the environment is meshed, the refined meshes are utilized to calculate the environment’s slope by extracting normal vectors from the surface geometry. This involves computing the orientation of the surface relative to a horizontal plane, which provides insights into the terrain’s topology and quantifies the steepness of the environment. This slope calculation facilitates better navigation and understanding of complex terrains.

Initially, the environment surface is represented as a refined mesh comprising vertices and faces, providing a comprehensive geometric model of the environment (see Section 3.3). Normal vectors for each face of the refined mesh at the centroid of each triangle are calculated through cross-product operations involving the edges of the face, providing information about the orientation of each terrain segment. A reference horizontal plane, defined along the XY-plane in a Cartesian coordinate system, is a flat, level surface extending infinitely in the X and Y directions with a constant Z-coordinate. It serves as the reference plane, with its normal vector pointing vertically upward along the Z-axis. By comparing the normal vector of each face to this reference plane, the angle of inclination is computed using trigonometric functions, giving a quantitative measure of the terrain’s steepness.

The slope $\alpha_{i}^{s}$ of i-th face is obtained using the dot product and magnitudes of the normal vectors (Equation (6)). Let $n_{i}^{s}$ be the normal vector of the i-th face at its centroid and $n^{ref}$ be the normal vector of the horizontal plane of the environment.

(6) $$\alpha_{i}^{s}=arccos\left(clip\left(\frac{n_{i}^{s}.n^{ref}}{\left\|n_{i}^{s}\right\|.\left\|n^{ref}\right\|},-1.0,1.0\right)\right)$$

To ensure numerical stability, the value is clipped to the range [−1, 1] before calculating the $\alpha_{i}^{s}$.

### 3.5. Centroid-Based Mesh and Roughness

By examining the meshing process for slope computation alongside roughness analysis, a detailed understanding of the terrain’s topology and surface characteristics is achieved. Surface roughness is an important parameter in fields such as geology, terrain analysis, and robotics, as it impacts the traversability of vehicles and robots on various terrains, making an understanding of surface roughness beneficial for applications like path-planning, geological mapping, and environmental monitoring. Roughness is quantified by analyzing spatial variations in elevation or slope, often relying on manual measurements or indirect proxies such as aerial imagery or LiDAR data [45,46].

In this paper, roughness is described as a measure of how uneven or irregular a surface is, determined by the angular deviation between neighboring normal vectors on a mesh-based representation of a surface. This approach helps quantify the level of irregularity in the terrain or surface. Using centroids of mesh triangles, described in Section 3.3, as vertices, a new alpha-shape triangular mesh, which we name the centroid-based alpha-shape mesh, is constructed, using the same procedure as that described in Section 3.3, allowing for the capture of local variations and irregularities in the environmental surface. This mesh is used to calculate terrain roughness by focusing on the angular deviation of adjacent centroids.

To calculate environment roughness $\beta_{i}^{r}$ for each face of the centroid-based alpha-shape mesh, the angular deviation between adjacent vertices is calculated. This process involves normalizing the normal vectors of the vertices, computing the dot products between adjacent normalized normal vectors of the vertices, and calculating roughness as the standard deviation of these dot products (Equation (7)). Consider a mesh represented by a set of vertices and faces. Let $F^{r}=\left\{f_{i}^{r}\;|\;i=1:n\right\}$ denote the set of faces in the centroid-based alpha-shape mesh, where each $f_{i}^{r}$ represents a face composed of three vertices. Additionally, let ${V^{r}=\{v}_{ij}^{r}\;|\;i=1:n,\;j=1:3\}$, with i as the index of the i-th face representing the set of vertices constituting the mesh. Let $N^{r}=\left\{n_{ij}^{r}\;\right|\;i=1:n,\;j=1:3\}$ denote the set of normal vectors corresponding to the vertices in $V^{r}$.

(7) $$\beta_{i}^{r}=std\left({\left\{\frac{n_{ij}^{r}}{\left\|n_{ij}^{r}\right\|}.\frac{n_{i\left(\left(j\%3\right)+1\right)}^{r}}{\left\|n_{i\left(\left(j\%3\right)+1\right)}^{r}\right\|}\right\}}_{j=1}^{3}\right)$$

The roughness value $\beta_{i}^{r}$ ranges between 0 and 1, with specific values indicating distinct surface characteristics. A roughness value of 0 signifies a perfectly flat surface, meaning that the normal vectors associated with the vertices of the face are perfectly aligned, showing minimal deviation from planarity. In contrast, a roughness value of 1 indicates a highly irregular or rough surface, where the normal vectors exhibit maximal misalignment, reflecting significant variations in surface orientation and substantial deviation from planarity. Intermediate values between 0 and 1 convey varying levels of surface irregularity, providing a continuous measure of surface roughness. While flat surfaces (low roughness) typically improve traversability, smooth surfaces (e.g., polished marble and ground glass) with low friction can reduce stability and pose safety risks, requiring careful consideration in robot path-planning and control strategies.

**Figure 4:**
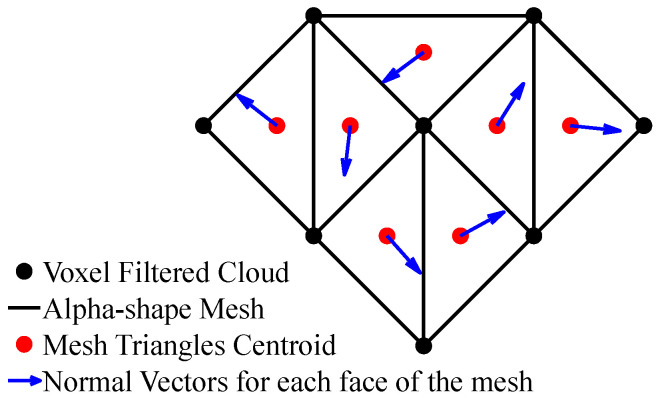
Methodology for calculating the slopeness of the environment.

Figure 4 illustrates the methodology for calculating the slopeness. A triangular alpha-shape mesh (black lines) is constructed from a voxel-filtered cloud (black dots). Subsequently, the centroid of each triangle (red dots) within the mesh is calculated. At these centroids, normal vectors are plotted. The angle between each of these normal vectors (blue vectors) and the normal vector of the horizontal plane of the environment provides the slopeness for each respective triangle.

**Figure 5:**
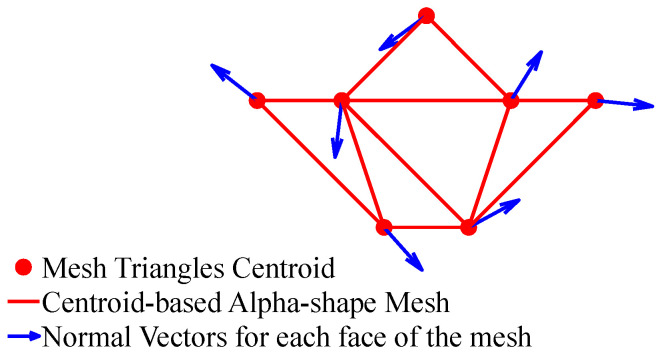
Methodology for calculating the roughness of the environment.

Building upon this, a centroid-based alpha-shape mesh is constructed using the centroids derived from the black triangles of the initial mesh. This secondary mesh, depicted in red lines, allows for calculating terrain roughness. The variation between the normal vectors established in the previous step is normalized to quantify the roughness across the surfaces of the red triangles (Figure 5).

This methodical approach allows for an assessment of terrain features by leveraging geometric constructions to deduce both slope and roughness from a given point cloud.

### 3.6. Virtual Box and Virtual Plane

In robotics, effective environment perception is crucial for safe and efficient traversing. This paper proposes a virtual box, a conceptual and dynamically defined boundary in 3D space positioned in front of the robot. The virtual box is defined at distance $d^{box}$ with dimensions of height $h^{box}$ and depth $l^{box}$. The distance $d^{box}$ between the robot and the virtual box is user-defined, ranging from the sensor’s minimum to maximum range. To reduce computational costs, it is recommended that the depth $l^{box}$ of the virtual box be determined based on the robot’s step size. The height $h^{box}$ is calculated using Equation (8).

(8) $$h^{box}=2\times d^{box}\times \tan\left(\frac{{fov}^{sensor}+R^{head}}{2}\right)$$

where ${fov}^{sensor}$ is the vertical field of view of the sensor and $R^{head}$ is the tilt range of the robot’s head. The virtual box moves along with the robot’s direction of motion and rotates according to the robot’s head orientation while maintaining the user-defined distance $d^{box}$.

The virtual box identifies whether the voxel-filtered cloud $p_{i}^{vfc}$ lies within it, indicating occupied spaces $p_{i}^{O}$ within the virtual box, thereby limiting the area of interest for data processing and analysis.

To enhance the computational processing time and interpretation of occupied space, a virtual plane, vp, is defined as the 2D representation of the virtual box, created by projecting the virtual box onto its front face, the side that faces the robot. The virtual plane is bounded by a rectangle defined by vertices $V^{vp}$. The vertices are given by $V^{vp}={\left\{\left(y_{i}^{vp},z_{i}^{vp}\right)\right\}}_{i=1}^{4}$, where $\left(y_{i}^{vp},z_{i}^{vp}\right)$ represents the vertices’ coordinates of the virtual plane.

The occupied spaces $p_{i}^{O}$ are projected onto the virtual plane, resulting in the 2D representation denoted as $p_{i}^{proj}=(y_{i}^{proj},z_{i}^{proj})$, which represents the coordinates of the occupied spaces within the virtual plane. This projection simplifies the data representation, allowing for a more efficient analysis and interpretation of the environment.

Overall, the definitions of the virtual box and virtual plane within the robotic perception system present a novel approach to environmental analysis. This methodology enhances computational efficiency and improves the robot’s ability to interpret complex environments. The continuous updates to the virtual plane enable the robot to maintain an up-to-date understanding of its surroundings, facilitating safer and more effective traversal. This work lays the foundation for future advancements in robotic navigation strategies, highlighting the crucial role of adaptive perception systems in real-world applications.

### 3.7. Find Empty Spaces on Virtual Plane

The virtual plane, established in Section 3.6, identifies occupied spaces $p_{i}^{proj}$ within its boundary. Any areas that remain unoccupied are considered empty spaces, representing regions that the robot can safely traverse. The virtual plane encapsulated within a bounding rectangle, provides a clear limit for the search area. This search area allows for a grid-based algorithm to divide the virtual plane into smaller cells, each serving as a candidate for identifying empty spaces.

The grid G is created by partitioning the virtual plane into cells of size G×G, where G is a user-defined parameter. A larger value of G results in fewer, larger cells, while a smaller value of G creates more, smaller cells. This parameter can be adjusted based on the specific requirements of the application, such as the desired resolution for identifying empty spaces or obstacles within the virtual plane.

To partition the virtual plane, calculating the number of rows and columns ensures that the virtual plane is divided appropriately based on its dimensions and the user-defined cell size G. The number of rows ($N_{r}^{G}$) and columns ($N_{c}^{G}$) in the grid G are calculated as Equation (9).

(9) $$N_{r}^{G}=\left\lceil \frac{\max\left(y_{i}^{vp}\right)-min(y_{i}^{vp})}{G}\right\rceil \;N_{c}^{G}=\left\lceil \frac{\max\left(z_{i}^{vp}\right)-\min\left(z_{i}^{vp}\right)}{G}\right\rceil$$

where ⌈ ⌉ denotes the ceiling function, which rounds any fractional result up to the nearest integer. This ensures that the grid cells fully cover the entire virtual plane.

Each grid cell is denoted as $g_{mn}$, where m represents the row index and n  represents the column index, within the grid G. The coordinates of each $g_{mn}$ within the vp are defined by Equation (10).

(10) $$y_{mn}^{G}=min\left(y_{i}^{vp}\right)+n.G\;z_{mn}^{G}=min\left(z_{i}^{vp}\right)+m.G$$

For each $g_{mn}$, the grid-based algorithm checks for intersections with occupied spaces by determining whether any $p_{i}^{proj}$ lie within the cell. If any points are found within the cell, it is marked as occupied; otherwise, it is considered empty. To designate empty spaces, the algorithm verifies two conditions for each grid cell $g_{mn}$ within the vp (Equation (11)).

(11) $$\left(m,n\right)\notin p_{i}^{proj}\;\left(m,n\right)\in vp$$

If both conditions are satisfied, the grid cell is considered an empty space. The set of all empty spaces within the grid is represented as vector $p_{i}^{E}$ (Equation (12)), where each element of the vector denotes a point in the cartesian coordinate system.

(12) $$p_{i}^{E}=\left\{\left(y_{i}^{E},z_{i}^{E}\right)\;\;|\;\;y_{i}^{E}\epsilon \;y_{mn}^{G},\;z_{i}^{E}\epsilon \;z_{mn}^{G},\;\neg \exists p_{i}^{proj}:\left(y_{i}^{E},z_{i}^{E}\right)\cap p_{i}^{proj}\neq \emptyset \right\}$$

The condition $\neg \exists p_{i}^{proj}:\left(y_{i}^{E},z_{i}^{E}\right)\cap p_{i}^{proj}\neq \emptyset$ denotes the logical negation of the existential quantifier, indicating the absence of any $p_{i}^{proj}$ intersecting with grid cell $g_{mn}$.

### 3.8. Robot’s Ellipsoidal Boundary and Determining Its Placement on the Virtual Plane

To evaluate whether a robot can effectively traverse its environment, a geometric representation of the robot facilitates the analysis and calculations related to its movement and interactions with the surroundings.

This paper introduces a methodology to determine ellipsoidal boundaries for robots based on their kinematics, motion state, and direction of motion, providing a mathematical representation that outlines the robot’s structure as an ellipsoid, providing a representation of the robot’s structures.

Determining a robot’s ellipsoidal boundary involves obtaining the robot’s primary dimensions—length, width, and height—from its kinematic model based on its configuration. The dimensions are influenced not only by the kinematic model but also by the robot’s current posture, such as walking or crawling. The specific configuration of the robot can lead to significant variations in the spatial arrangement of its components, thereby affecting the measurements of length, width, and height. For instance, when the robot is standing upright, its height (Z-axis) is maximized, reflecting the full vertical extent of its structure. In contrast, when the robot transitions to a crawling position, its height decreases, and the positions of its limbs shift, impacting the overall length (X-axis) and width (*Y*-axis). During dynamic movements like walking or running, the extension and retraction of the arms and legs can lead to further changes in these effective dimensions. Similarly, while crawling, the orientation of the limbs alters significantly, often bringing the torso closer to the ground and changing the maximum and minimum coordinates used to define its dimensions.

This approach allows for a more precise representation of the robot’s geometry in its operational environment, ensuring that the ellipsoidal boundary effectively encapsulates the robot’s spatial characteristics across various movements and configurations.

To obtain each robot’s dimension, the maximum and minimum coordinates along their respective axes (X, Y, and Z) are calculated, capturing the robot’s furthest extents within the context of its configuration. These dimensions represent the length, width, and height of the robot. The robot’s dimensions in the sagittal, median, and frontal planes are represented as a vector, denoted as ${size}^{robot}=(x_{size}^{robot},y_{size}^{robot},z_{size}^{robot})$.

The ellipsoid is defined using parametric equations, with the semi-axes lengths calculated as ${a^{el}=x}_{size}^{robot}/2$, $b^{el}=y_{size}^{robot}/2$, and $c^{el}=z_{size}^{robot}/2$, along with center coordinates $C^{el}=\left(x_{C}^{el},y_{C}^{el},z_{C}^{el}\right)$ (see Equation (13)). The centroid of the ellipsoid $C^{el}$ calculated by averaging the maximum and minimum coordinates along the X, Y, and Z axes. The maximum and minimum values correspond to the robot’s furthest extents (for example, the position of the robot’s head and feet along the Z-axis, and similarly for the X and Y axes).

(13) $$\begin{array}{c} x_{u,v}^{el}=x_{c}^{el}+a^{el}cos\left(u\right)sin\left(v\right)\\ y_{u,v}^{el}=y_{c}^{el}+b^{el}sin\left(u\right)sin\left(v\right)\\ z_{u,v}^{el}=z_{c}^{el}+c^{el}cos\left(v\right) \end{array}$$

where the variable $u\;\epsilon \;\left[0,\;2\pi \right]$ and the variable $v\;\epsilon \;\left[0,\;\pi \right]$. These parametric equations generate a uniformly distributed set of points across the surface of the ellipsoid, with u representing the azimuthal angle and v the polar angle.

Once the ellipsoidal boundary encapsulating the robot is determined, the next step is to project it onto the virtual plane. This paper introduces a method for determining the placement of the projection of the robot’s ellipsoidal boundary—an ellipse—onto the virtual plane. The objective is to position the ellipse within the virtual plane in such a way that it avoids intersecting any occupied cells of grid G. To achieve this, a grid-based search algorithm is employed to place the ellipsoid boundary within the virtual plane.

Initially the algorithm attempts to place an ellipse along the direction of the robot’s movement, within the grid G. The placement is verified by checking that no part of the ellipse intersects any occupied cells within grid G. This is performed using an inequality to determine whether any $p_{i}^{proj}$ lies inside the ellipse, as expressed in Equation (14).

(14) $$\frac{{\left(y_{i}^{proj}-y_{C}^{el}\right)}^{2}}{{b^{el}}^{2}}+\frac{{\left(z_{i}^{proj}-z_{C}^{el}\right)}^{2}}{{c^{el}}^{2}}\leq 1$$

If any $p_{i}^{proj}$ satisfies this inequality, this indicates an intersection with an occupied cell, and the placement of the ellipse at that location is invalid. However, if this condition is not satisfied, the ellipse is stored as a candidate ellipse.

The search for valid placements continues by evaluating neighboring empty cells in grid G. Immediate neighboring empty cells are checked first, followed by an extended search over wider grid cells. Neighboring cells are defined as the set of positions obtained by shifting the current centroid of the ellipse on grid G, as expressed in Equation (15). These shifts occur in all possible directions (e.g., up, down, left, right, and diagonally). The new centroids are calculated based on the initial centroid $C_{0}^{el}$, denoted as $\left(y_{C}^{el},z_{C}^{el}\right)$, and the shifts $\left(dy,dz\right)$, where dy and dz take values from $\left\{\pm G\right\}$ (representing the grid size G). This process continues, defining a new centroid with each shift, until all empty cells, $p_{i}^{E}$, have been evaluated.

(15) $$C_{i}^{el}=\left\{C_{0}^{el}+\left(dy,dz\right)|dy,dz\in \left\{\pm G\right\},p_{i}^{E}\right\}$$

For each empty cell, the same inequality (Equation (14)) is applied. If no intersections are detected, the ellipse positioned at that cell is stored in a set of candidate ellipses.

Selecting an appropriate ellipse between candidate ellipses within the virtual plane emphasizes the need for a scoring metric method that incorporates three score metrics: fit to a virtual plane, avoidance of obstacles, and proximity to the ground. The methodology outlined herein formulates each component of the scoring system mathematically, allowing for a comprehensive evaluation of potential ellipse placement.

The score metric $S_{n}^{el}$ for the n-th candidate ellipse within the virtual plane is calculated as Equation (16).

(16) $$S_{n}^{el}=F_{n}^{el}-A_{n}^{el}-G_{n}^{el}$$

where $F_{n}^{el}$ is the fit score, $A_{n}^{el}$ is the avoidance score, and $G_{n}^{el}$ is the proximity-to-ground score. The fit score $F_{n}^{el}$, Equation (17), evaluates how well an ellipse fits within the virtual plane. It is calculated by evaluating the boundaries of the ellipse relative to the defined limits of the virtual plane ($y_{min}^{vp}$, $y_{max}^{vp}$, $z_{min}^{vp}$, $z_{max}^{vp}$).

(17) $$F_{n}^{el}=max\left(0.1-\frac{D_{n}^{el}}{L_{n}^{el}}\right)$$

where $D_{n}^{el}$ is the distance of candidate ellipse boundary from the virtual plane boundary, calculated as Equation (18). $L_{n}^{el}$ is the sum of the semi-axes lengths of a candidate ellipse (Equation (19)).

(18) $$D_{n}^{el}=\left(b^{el}-min\left(y_{max}^{vp}-y_{C}^{el},y_{C}^{el}-y_{min}^{vp}\right)\right)+\left(c^{el}-min\left(z_{max}^{vp}{-z}_{C}^{el},{z_{C}^{el}-z}_{min}^{vp}\right)\right)$$

(19) $$L_{n}^{el}=b^{el}+c^{el}$$

The avoidance score metric, Equation (20), evaluates how well the ellipsoid avoids obstacles in the virtual plane. This score metric is calculated by evaluating the proximity of the ellipsoid to any identified $p_{i}^{proj}$. The method checks whether any candidate ellipse falls within a safety margin with radius δ around each obstacle.

(20) $$A_{n}^{el}=\sum_{j=1}^{i}P_{i}$$

where $P_{i}$ is a penalty for each obstacle i. If the ellipse is deemed too close to an obstacle (within the safety margin), the penalty is set as $P_{i}=1.0$. The total avoidance score accumulates these penalties for all overlapping obstacles.

The proximity-to-ground score $G_{n}^{el}$ quantifies how close a candidate ellipse is to the ground surface. A lower score indicates a better position closer to the ground, facilitating stability during navigation. The proximity-to-ground score $G_{n}^{el}$ is defined based on the vertical distance between the lowest part of the ellipse, ($z_{C}^{el}-c^{el}$), and the ground, $z_{i}^{proj}$ (see Equation (21). A lower score metric indicates that the ellipse is positioned closer to the ground, which is often desirable for stability and effective navigation.

(21) $$G_{n}^{el}=\left(z_{C}^{el}-c^{el}\right)-max\left(z_{i}^{proj}\right)$$

**Figure 6:**
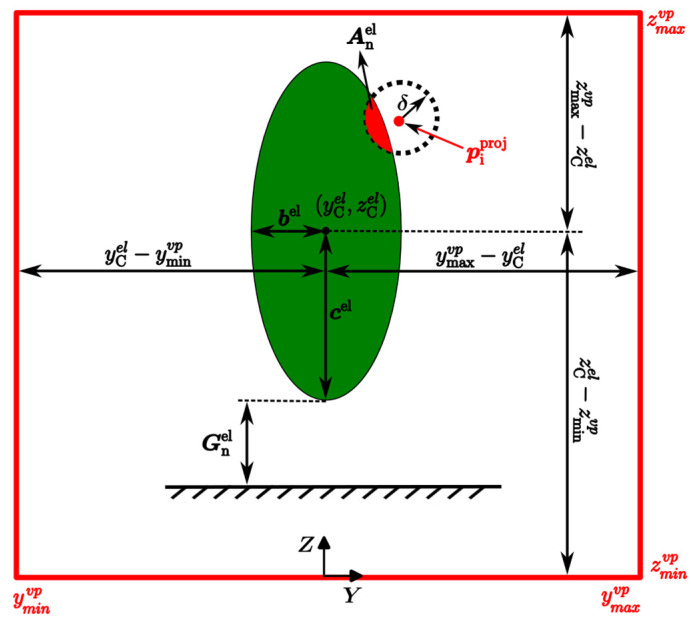
The score metric $S_{n}^{el}$ for the n-th candidate ellipse within the virtual plane.

Figure 6 illustrates the evaluation metrics used to score candidate ellipse within the virtual plane, defined by the boundaries $y_{min}^{vp}$, $y_{max}^{vp}$, $z_{min}^{vp}$, and $z_{max}^{vp}$. The candidate ellipse, characterized by its center $\left(y_{C}^{el},z_{C}^{el}\right)$ and semi-axes’ lengths $b^{el}$ and $c^{el}$, is shown fitting within the plane. The fit score $F_{n}^{el}$ evaluates how well the green ellipse fits within the virtual plane, considering the distances between its boundaries and the virtual plane limits (red rectangle). The avoidance score $A_{n}^{el}$ is represented by the proximity of the ellipse to any obstacle ($p_{i}^{proj}$).

As depicted in the picture, for each obstacle ($p_{i}^{proj}$, shown as a red dot), a circular safety margin or “halo” with radius δ (dashed circle) is defined. This safety margin acts as a buffer zone around the obstacle, and if any part of the ellipse overlaps with this margin, as indicated by the red-colored region inside the safety margin, a penalty is assigned. The proximity-to-ground score $G_{n}^{el}$ favors ellipses positioned closer to the ground. In the figure, the vertical distance from the lowest point of the ellipse ($z_{C}^{el}-c^{el}$) to the ground level ($z_{i}^{proj}$) is illustrated.

## 4. Results

### 4.1. Case Study

The Transcend humanoid robot used to test the proposed SRE indices features 37 degrees of freedom (DOFs), providing it with a broad range of movements and interactions within its environment. It is equipped with advanced sensors and actuators, including a Velodyne VLP-16 LiDAR for environmental scanning and an Intel RealSense T265 Camera for real-time tracking and navigation. The robot’s movement capabilities are enhanced by Dynamixel actuators, allowing it to handle complex tasks. Data acquisition and calculations are performed on a laptop with an 11th Gen Intel^®^ Core™ i7-11800H CPU (2.30 GHz, 16 cores) and 16 GB of memory. The primary objective was to evaluate the robot’s ability to perceive and interpret various environmental features, which is crucial for navigating through different settings, particularly those with complex surfaces or obstacles.

**Figure 7:**
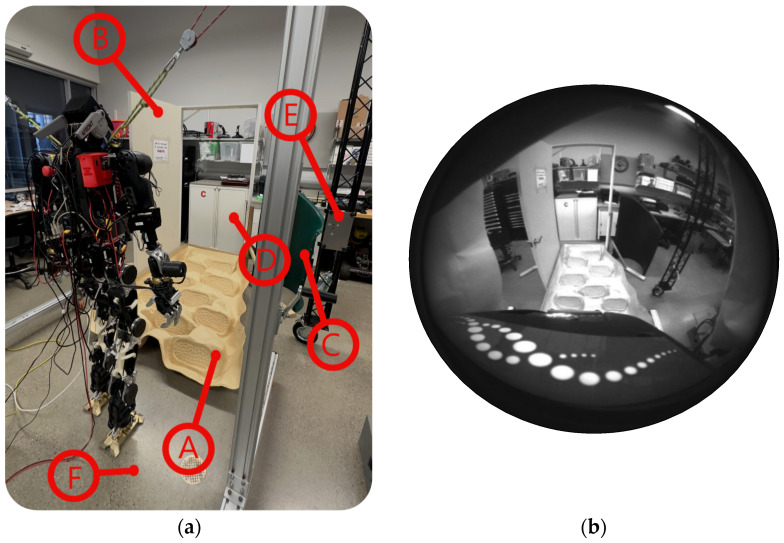
(**a**) The space directly ahead of the robot (A: Uneven surface, B: Door, C: Curved rigid body, D: Closet, E: Structured truss, F: Flat surface). (**b**) The surroundings recorded by the camera mounted on the robot’s head.

During the test, the robot used its LiDAR sensor to scan the environment and detect traversable pathways. The test scenario positioned the robot in an area featuring a mix of uneven and flat terrains with obstacles, as depicted in Figure 7a.

The robot continuously observed its surroundings through its camera, as shown in Figure 7b. This setup demonstrates the proposed SRE indices’ ability to operate effectively in semi-structured environments where precise environmental perception is crucial for successful task execution. It highlights the SRE indices’ potential applications in real-world tasks involving diverse terrain and unexpected obstacles.

The experimental test was conducted in semi-structured environments, which provided varying degrees of complexity for assessing the proposed approach. To address the high uncertainty encountered in outdoor, unstructured terrains, further enhancements may be required. This paper provides a foundational stepping stone for such advancements, enabling future research to enhance and extend the proposed approach.

### 4.2. Evaluation of Point Cloud Data Refinement Through Voxel-Filtering

The point cloud data, captured by the LiDAR sensor, represented the surrounding environment of the robot in 3D space. The Velodyne VLP-16 LiDAR sensor offers a typical range accuracy of up to ±3 cm, a vertical field of view of +10.0° to −10.0° (20°), and angular resolutions of 1.33° vertically and between 0.1° to 0.4° horizontally.

The raw point cloud data captured by the sensor were transformed from the sensor’s reference frame to the robot’s base frame using a homogeneous transformation matrix; this was followed by voxel-filtering, resulting in a refined representation of the point cloud.

**Figure 8:**
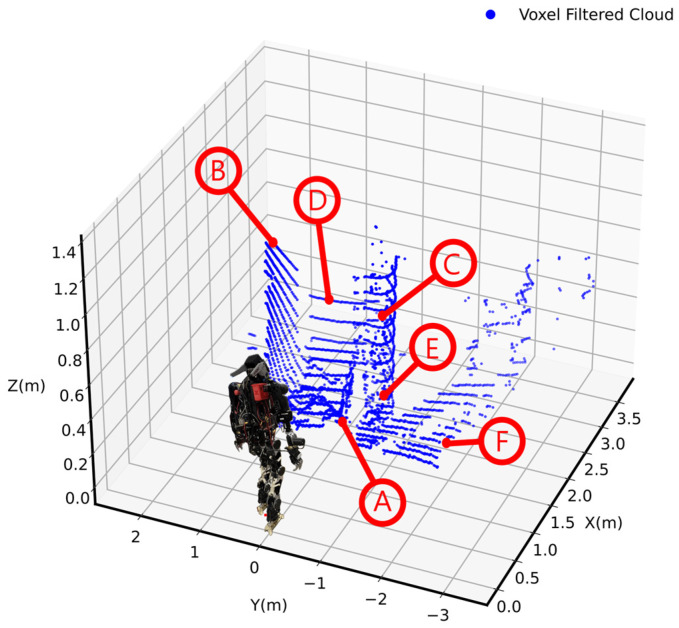
Voxel-filtered cloud (A: Uneven surface, B: Door, C: Curved rigid body, D: Closet, E: Structured truss, F: Flat surface).

The performance of the voxel-filtering technique was evaluated based on the point cloud data collected by the LiDAR sensor. The raw point cloud data obtained from the sensor $p_{i}^{raw}$ comprised 3585 points, subsequently reduced to 3028 points after applying transformation and voxel-filtering (see Figure 8). This reduction of 557 points indicated that approximately 15.6% of the $p_{i}^{raw}$ was removed. The points filtered out were primarily attributed to noise and redundancy, as voxel-filtering aggregates points within defined voxel sizes and retains only unique representatives. As a result, points resulting from sensor noise, such as those arising from environmental reflections or inaccuracies, were likely to be among those filtered out. This reduction indicated the effectiveness of the filtering process in removing redundant points while preserving crucial spatial information, thereby enhancing computational efficiency for further processing steps.

The mean distance between the transformed points, $p_{i}^{t}$, and their nearest voxel counterparts was calculated as 0.0097 m, indicating that the voxel-filtered points closely approximated the original spatial data. Although the maximum distance of 0.0163 m indicated that some points were not perfectly matched, they remained within an acceptable range. Therefore, the filtering process maintained spatial integrity. The minimum distance of 0.0007 m highlighted that certain points were almost identical after filtration.

In summary, the application of voxel-filtering resulted in a significant reduction in point count while effectively preserving the accuracy of the spatial representation. The refined point cloud data were essential for improving the robot’s perception capabilities, facilitating more accurate environmental modeling and analysis.

### 4.3. Evaluation of Environment Mesh

**Figure 9:**
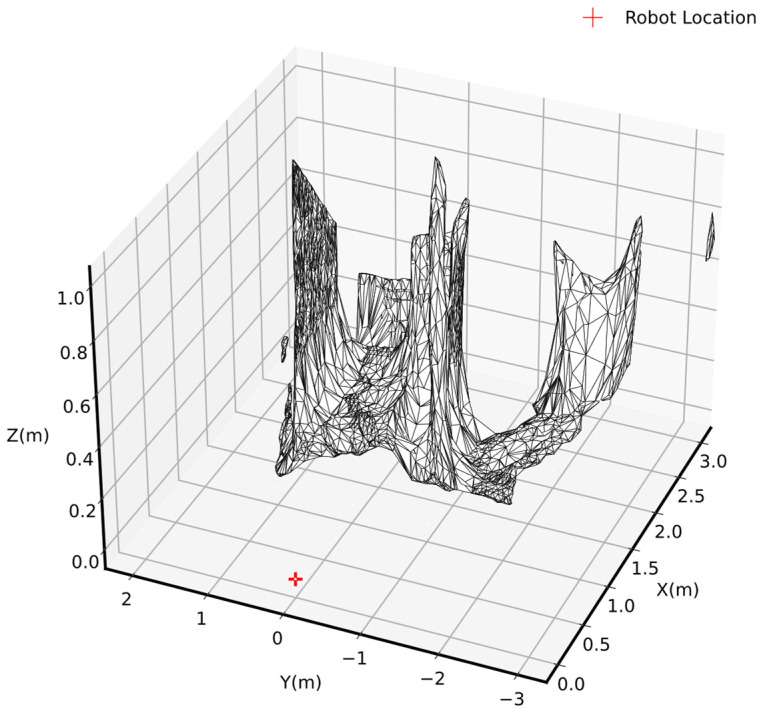
Environment’s mesh structured using alpha shapes method.

The image in Figure 9 depicts the result of applying a meshing algorithm to the voxel-filter point cloud. The mesh analysis generated from the voxel-filter cloud data provided valuable insights into the geometric properties of the evaluated surfaces. By employing the alpha shape algorithm, the voxel-filtered data were processed to extract a mesh representation of the environment. The analysis of the generated mesh revealed several key characteristics related to its geometric properties and surface features.

The processing of the voxel-filtered points resulted in a total of 4750 faces, contributing to the mesh’s overall structure. The total surface area of the mesh was determined to be 15.678 m^2^. The triangle area statistics provided more detail on the mesh geometry, with a minimum area of 0.000200 m^2^, a maximum of 0.044429 m^2^, a mean of 0.003301 m^2^, and a standard deviation of 0.004871 m^2^. These statistics indicated a range of triangle sizes, reflecting the complexity of the surface being modeled. The standard deviation, compared to the mean value, indicated that the size of triangles varied. This comparison suggested that the mesh had a non-uniform resolution, with some triangles containing fine areas (smaller triangles) and other regions with coarser areas (larger triangles).

In general, mesh analysis is a preparatory step toward the further analysis of surface geometric and physical properties. Consequently, the extracted mesh provided a comprehensive overview of triangle distribution, surface characteristics, and area metrics, forming the basis for calculating slopeness and roughness.

### 4.4. Evaluation of Environment Slopeness

**Figure 10:**
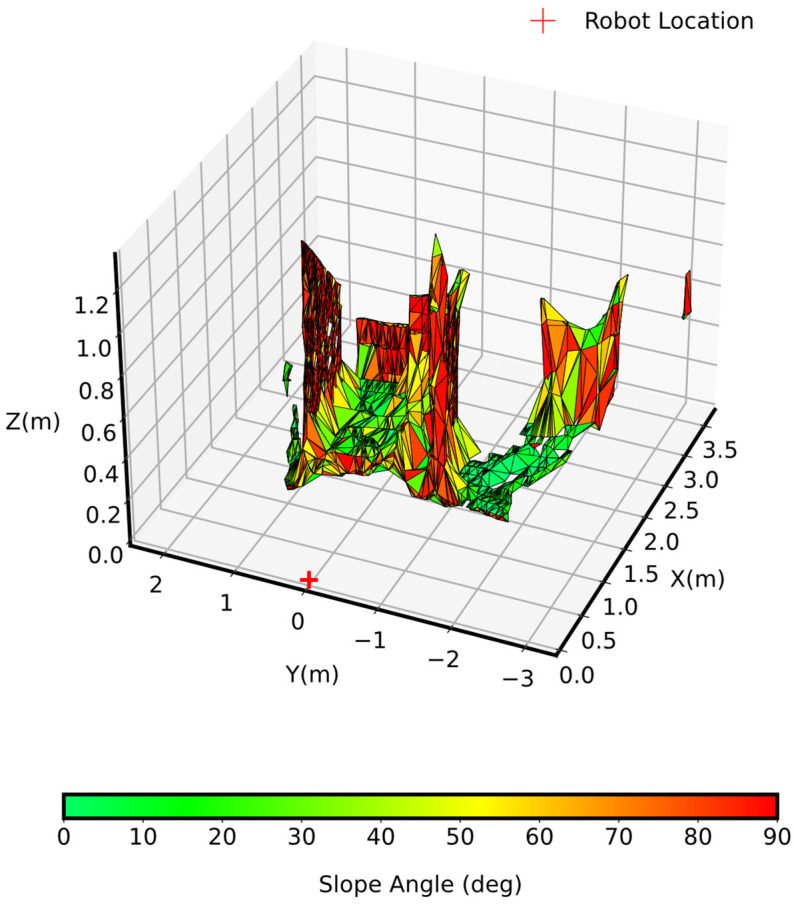
Slopeness of the environment.

The slopeness of the terrain as depicted in the 3D plot in Figure 10 illustrated the slopeness of the terrain derived from the generated mesh, providing a comprehensive overview of the environment’s gradients. This was crucial for assessing the robot’s capability to traverse various parts of the environment and identify potential risk areas that might hinder movement.

The slopeness is here represented in colors, ranging from green for flat terrains (having a 0° slope) to red for steep terrains, providing a visual representation of the slopeness of the terrain. The green areas indicate flat terrain, ideal for safe robot traversal, whereas orange and red zones indicate challenging terrain that may require cautious maneuvering or avoidance. The analysis of the mesh indicated that only 27% of the area had slopeness of less than 10°, 12% had slopeness between 10° and 30°, while 61% of the overall area had slopeness greater than 30°.

Areas with slopeness ranging from 0° to 10° facilitate faster traversal. In moderate sloped terrain which ranges from 10° to 30°, adjusting speed or changing the configuration style can enhance stability. The areas with slopeness above 30° represent challenging terrain where a robot may need to change its locomotion style (e.g., switching from walking to crawling) to traverse effectively. Overall, areas with the least slopeness are generally right for high speed and safe traversal, whereas slopeness that is steep requires slow and careful movements to remain stable.

### 4.5. Evaluation of Environment Roughness

**Figure 11:**
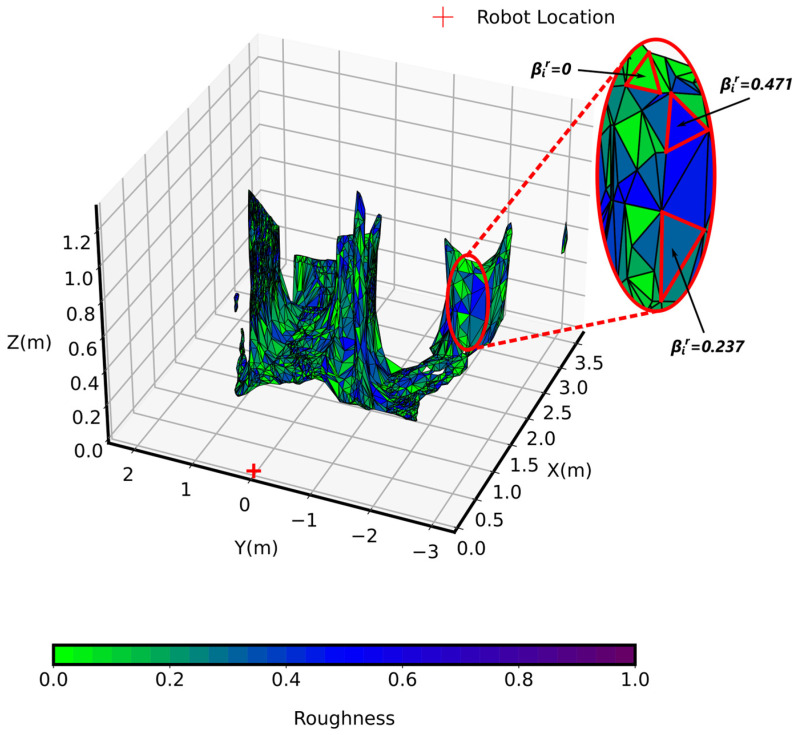
Roughness of the environment.

The image in Figure 11 depicts the roughness of the environment, an important factor in evaluating a robot’s traversability and ability to maintain effective movement across different surfaces.

Surface roughness was analyzed using a centroid-based mesh approach, which captured local variations and irregularities in the terrain. This method enabled us to compute roughness by examining the angular deviations between the normal vectors of adjacent vertices on a mesh-based representation of the surface.

The roughness is color-coded here, ranging from green (indicating flat terrain) to purple (which represents areas of high roughness where the angular deviation between adjacent vertices $v_{ij}^{r}$ in each face $f_{i}^{r}$ was significant). This color coding provides an intuitive visual representation of terrain roughness, giving an easy way of identifying the surface characteristics that the robot could encounter. Roughness values were assigned to each face of the mesh, with values ranging from 0 to 1, with 1 representing high irregularity and 0 representing perfectly flat surfaces.

Green areas with roughness values near 0 indicated flat surfaces that were easy to navigate, while purple areas where roughness approached 1 represented rough surfaces, such as rocky or uneven ground. These purple areas signified surfaces that were difficult to traverse.

The evaluation of environment roughness provided several important statistical characteristics, which are summarized in Table 1. The mean roughness value was roughly 0.237, indicating a moderate level of roughness that could affect the robot’s motion. The standard deviation of the roughness was found to be 0.139, suggesting a moderate variability in the roughness measurements. This relatively low standard deviation compared to the mean indicated that while the terrain variability was noticeable regarding roughness characteristics, the roughness variations were relatively modest. The maximum observed roughness value was 0.471, indicating the presence of significantly rough terrain features that could be challenging to traverse over. In contrast, the minimum roughness was equal to 0, indicating the presence of completely flat surface areas ideal for efficient maneuvering.

The roughness analysis was crucial for advanced path-planning and enabled the robot to adapt to the different surface geometry. For instance, the robot could reduce its speed or be more cautious when moving from green areas to purple areas, improving operational efficiency. However, areas with $\beta_{i}^{r}=0$ flat surfaces could still exhibit challenges due to low friction. While a roughness value of $\beta_{i}^{r}=0$ indicated a flat surface with minimal geometric irregularities, this did not necessarily imply high traction. Flat surfaces such as polished marble or ground glass, despite having low roughness, may be smooth and slippery, posing stability challenges. Therefore, while roughness plays a critical role in assessing terrain traversability, smoothness remains an important factor to be considered, particularly when assessing traction and stability in real-world applications. This highlights the need for future work to incorporate frictional properties in combination with roughness for a more complete analysis of traversability.

### 4.6. Virtual Box and Virtual Plane Analysis

**Figure 12:**
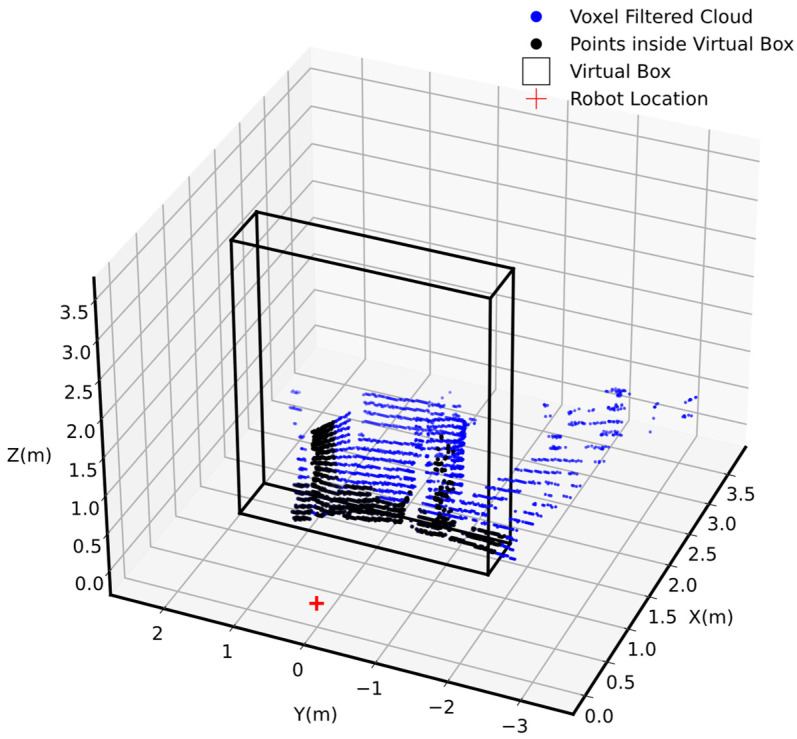
Virtual box in front of the robot.

As illustrated in Figure 12, the proposed virtual box limits the area of interest in front of the robot for subsequent data processing and analysis. The virtual box identified the voxel-filtered cloud, $p_{i}^{vfc}$ (shown in blue), within it, allowing for a more focused analysis of the environment. The identified points within the virtual box, representing occupied spaces, $p_{i}^{O}$, indicated obstacles and terrain features that could affect the robot’s movement.

**Figure 13:**
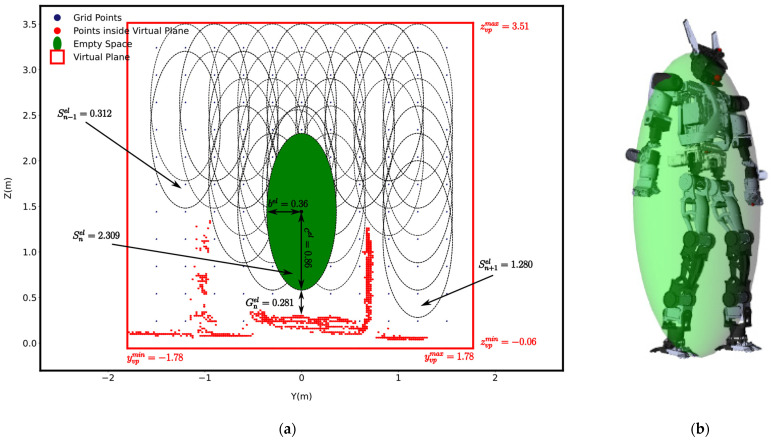
(**a**) Placing the projection of ellipsoid boundary onto the virtual plane. (**b**) Robot’s ellipsoid boundary.

Figure 13a shows the voxel-filtered cloud, $p_{i}^{proj}$ (red dots), projected onto the virtual plane (red rectangle). This projection showed the location and distribution of obstacles in the virtual plane. The virtual plane facilitated the analysis of the space in front of the robot, reducing the computational load and improving processing speed for subsequent analysis.

### 4.7. Identifying Empty Spaces on the Virtual Plane

The methodology described in Section 3.7 effectively identifies empty spaces within a virtual plane by employing a grid-based algorithm (see Figure 13a). This approach partitioned the virtual plane into cells, identifying occupied and empty spaces. If any points (red points) were found within the cell, it was marked as occupied; otherwise, it was considered empty.

### 4.8. Creation and Adaptation of the Robot’s Ellipsoidal Boundary

Figure 13b illustrates the robot encapsulated within an ellipsoid boundary (green ellipsoid). The ellipsoid’s size changed according to the robot’s configuration. It expanded or contracted as the robot’s configuration changed (e.g., extended its arms) to account for the space the robot occupied. The ellipsoid boundary dynamically adapted to allow the robot to be aware of how much space it required at any moment for safe traversal through its environment.

### 4.9. Fitting the Projection of Ellipsoid Boundary onto the Virtual Plane

Figure 13a demonstrates the placement of the projection of the robot’s ellipsoid boundary onto the virtual plane. The grid-based search algorithm effectively identified a place to fit the projected ellipsoid boundary, shown as the green ellipsoid. This visualization clearly distinguished between traversal and non-traversal areas, allowing the robot to make a decision about its navigation path.

The red points in the Figure 13a represent obstacles or occupied areas that the robot needed to avoid and the blue points represent grid points used by the algorithm to search for potential ellipse placements.

The score metric $S_{n}^{el}$ evaluated candidate ellipses, shown by the dashed ellipses. The figure specifies the $S_{n}^{el}$ of three candidate ellipses, with $S_{n-1}^{el}=0.312$, $S_{n}^{el}=2.309$, and $S_{n+1}^{el}=1.280$. Among these candidates, the green ellipse represents the selected ellipse, which was chosen based on achieving the maximum score of $S_{n}^{el}=2.309$. The scoring components for the selected ellipse were as follows:

- Fit score ($F_{n}^{el}$): 2.59
- Avoidance score ($A_{n}^{el}$): 0
- Proximity-to-ground score ($G_{n}^{el}$): 0.281

To clarify the evaluation process and interpretation of Figure 13a, the scoring components for the selected ellipse were calculated as follows.

- The fit score $F_{n}^{el}$ was calculated using Equation (16)

(22) $$\begin{array}{c} D_{n}^{el}=\left(b^{el}-min\left(y_{max}^{vp}-y_{C}^{el},y_{C}^{el}-y_{min}^{vp}\right)\right)+\left(c^{el}-min\left(z_{max}^{vp}{-z}_{C}^{el},{z_{C}^{el}-z}_{min}^{vp}\right)\right)\\ \;\;\;\;\;\;=\left(0.36-min\left(1.78-0,\;0-(-1.78\right)\right)+\left(0.86-min\left(3.51-1.44,\;1.44-(-0.06\right)\right)\\ \;\;\;\;\;\;=-1.94 \end{array}\;\begin{array}{c} L_{n}^{el}={b^{el}+c}^{el}=0.36+0.86=1.22\\ \;F_{n}^{el}=max\left(0.1-\frac{-1.94}{1.22}\right)=2.59 \end{array}$$

- The avoidance score $A_{n}^{el}$ was equal to zero since no part of the selected ellipse overlapped with safety margin of obstacle ($p_{i}^{proj}$).
- The proximity-to-ground score $G_{n}^{el}$ was calculated using Equation (20):

(23) $$G_{n}^{el}=\left(z_{C}^{el}-c^{el}\right)-max\left(z_{i}^{proj}\right)=\left(1.44-0.86\right)-0.299=0.281$$

- The score metric $S_{n}^{el}$ for the selected ellipse within the virtual plane was calculated as Equation (15):

(24) $$S_{n}^{el}=F_{n}^{el}-A_{n}^{el}-G_{n}^{el}=2.59-0-0.281=2.309$$

This method allowed the system to determine whether the robot could proceed through specific paths or if adjustments were needed to avoid obstacles. This can allow robots to operate in dynamic environments.

### 4.10. Applicability to Path-Planning (Future Work)

Path-planning is a crucial aspect of autonomous robot navigation, enabling robots to determine optimal paths through complex environments. The SRE indices proposed in this paper provide valuable information for path-planning by quantifying key terrain characteristics. By evaluating the traversability of different terrain regions, this method can directly inform path-planning algorithms and improve navigation performance. In path-planning applications, the SRE indices can be used to create a cost map that assigns higher traversal costs to less navigable areas.

For example, graph-based planners such as A* or D* can incorporate these costs into their path evaluation functions to prioritize routes through safer, more efficient regions. Similarly, sampling-based planners like RRT* or PRM can leverage these scores to avoid sampling paths in highly challenging or non-traversable areas. In dynamic or uncertain environments, the traversability assessment can also enable adaptive path-planning. By updating the cost map in real time, a robot can adjust its planned trajectory to account for unexpected changes in the terrain, ensuring safe and effective navigation.

It is important to note that while this paper focuses on developing and evaluating terrain traversability, the implementation of a full path-planning system is outside its scope. However, the presented approach lays the groundwork for such applications, providing a valuable tool for future research and development in autonomous navigation.

## 5. Conclusions

This paper presented a comprehensive approach for evaluating robot traversability in complex terrains through the use of slopeness–roughness–empty space (SRE) indices. The proposed approach addresses the key challenges posed by unstructured environments, such as uneven surfaces, obstacles, and varying terrain surfaces, which often hinder the performance of robots in real-world applications like disaster recovery and search-and-rescue missions. These indices improve robot perceptions of dynamic environments, thus improving their ability to move safely and efficiently across various terrains.

Our experimental results, conducted with the Transcend humanoid robot featuring 37 degrees of freedom (DOFs), validated the effectiveness of the SRE indices in a mixed-terrain test scenario. Through visualizations and quantitative evaluations, we demonstrated that regions with low slopeness and roughness are optimal for rapid traversal, while areas with steeper inclines or greater surface irregularities necessitate caution and may require a transition in locomotion, such as switching from walking to crawling.

Furthermore, the virtual box and virtual plane reduced the computational load and improved processing speed for subsequent analysis by limiting the areas of interest in front of the robot. The ellipsoidal boundary adapted dynamically to the robot’s posture to prevent collision and enable effective movement. Projection of the ellipsoid onto the virtual plane using a grid-based search algorithm identified traversable areas and helped the robot determine how to traverse the area safely.

In summary, the SRE indices and the associated processing framework presented in this paper offer a comprehensive solution for improving robot navigation in semi-structured environments. This methodology enables autonomous systems to operate safely and efficiently in semi-structured terrains by utilizing advanced point cloud processing techniques integrating voxel-filtered cloud, boundary and mesh generation, and dynamic traversability.

## Figures and Tables

**Table 1:** Statistical evaluation of the environment roughness.

Statistical Evaluation	Value	Description
Mean Roughness	0.237	Represents a moderate level of terrain roughness that may influence a robot’s movement.
Standard Deviation of Roughness	0.139	Indicates moderate variability in terrain roughness.
Maximum Roughness	0.471	Reflects areas with notably rough terrain that could challenge a robot’s traversal.
Minimum Roughness	0	Denotes flat surfaces, ideal for optimal robot movement and efficiency.

## Data Availability

Data is contained within the article.
